# Structural and biochemical analyses of alanine racemase from the multidrug-resistant *Clostridium difficile* strain 630

**DOI:** 10.1107/S1399004714009419

**Published:** 2014-06-29

**Authors:** Oluwatoyin A. Asojo, Sarah K. Nelson, Sara Mootien, Yashang Lee, Wanderson C. Rezende, Daniel A. Hyman, Monica M. Matsumoto, Scott Reiling, Alan Kelleher, Michel Ledizet, Raymond A. Koski, Karen G. Anthony

**Affiliations:** a National School of Tropical Medicine, Department of Pediatrics, Baylor College of Medicine, Houston, TX 77030, USA; b L^2^ Diagnostics LLC, 300 George Street, New Haven, CT 06511, USA; c Pathology and Microbiology Department, University of Nebraska Medical Center, Omaha, NE 68105, USA

**Keywords:** *Clostridium difficile* infection, alanine racemase, pyridoxal-5′-phosphate, drug development, multidrug resistance, nosocomial infections, cycloserine, structure-based drug design

## Abstract

*Clostridium difficile*, a Gram-positive, spore-forming anaerobic bacterium, is the leading cause of infectious diarrhea among hospitalized patients. *C. difficile* is frequently associated with antibiotic treatment, and causes diseases ranging from antibiotic-associated diarrhea to life-threatening pseudo­membranous colitis. The severity of *C. difficile* infections is exacerbated by the emergence of hypervirulent and multidrug-resistant strains, which are difficult to treat and are often associated with increased mortality rates. Alanine racemase (Alr) is a pyridoxal-5′-phosphate (PLP)-dependent enzyme that catalyzes the reversible racemization of l- and d-alanine. Since d-alanine is an essential component of the bacterial cell-wall peptidoglycan, and there are no known Alr homologs in humans, this enzyme is being tested as an antibiotic target. Cycloserine is an antibiotic that inhibits Alr. In this study, the catalytic properties and crystal structures of recombinant Alr from the virulent and multidrug-resistant *C. difficile* strain 630 are presented. Three crystal structures of *C. difficile* Alr (CdAlr), corresponding to the complex with PLP, the complex with cycloserine and a K271T mutant form of the enzyme with bound PLP, are presented. The structures are prototypical Alr homodimers with two active sites in which the cofactor PLP and cycloserine are localized. Kinetic analyses reveal that the K271T mutant CdAlr has the highest catalytic constants reported to date for any Alr. Additional studies are needed to identify the basis for the high catalytic activity. The structural and activity data presented are first steps towards using CdAlr for the development of structure-based therapeutics for *C. difficile* infections.

## Abstract 2

Structures of *C. difficile* alanine racemase in complex with the PLP cofactor and cycloserine are presented.

## 1. Introduction   

The Gram-positive spore-forming anaerobic bacterium *Clostridium difficile* was discovered in the feces of newborns by Hall and O’Toole in 1935, and was initially named *Bacillus difficile* owing to the difficulty of culturing it *in vitro* (Hall & O’Toole, 1935 ▶). The multiple strains of *C. difficile* are either dormant spore-forming or vegetative toxin-producing forms (Bartlett, 2009*a* ▶). Both forms cause disease; however, the spore-forming forms are resistant to antibiotics while the vegetative forms are more susceptible to some antibiotics (Bartlett, 2009*b* ▶). *C. difficile* was identified in 1978 as the agent responsible for pseudomembranous colitis, with hypervirulent strains, including strain 630, causing morbidity and mortality after exposure of patients to fluoroquinolinones or proton-pump inhibitors (Carroll & Bartlett, 2011 ▶). *C. difficile* is a troubling nosocomial bacterium that is known to contaminate hospital air, surfaces and personnel, thus posing a great risk to immunocompromised patients (Carroll & Bartlett, 2011 ▶; Best *et al.*, 2010 ▶). Drug-resistant *C. difficile* infections usually result in explosive diarrhea, exacerbating the spread of the bacteria in hospitals and the community (Burnham & Carroll, 2013 ▶; Best *et al.*, 2010 ▶; Bartlett, 2009*a* ▶). The virulence and mortality of *C. difficile* infection is on the rise; thus, there is an urgent need for new therapeutics to combat this pathogen (Burnham & Carroll, 2013 ▶).

Alanine racemase (Alr; EC 5.1.1.1) is the prototypical member of the fold type III superfamily of enzymes that are dependent on pyridoxal-5′-phosphate (PLP) for catalysis (Jansonius, 1998 ▶). Alr catalyzes the reversible racemization of l-alanine and d-alanine; the latter is a key building block in the biosynthesis of the peptidoglycan layer in bacterial cell walls (Walsh, 1989 ▶). Alr has been extensively studied as a target for antimicrobial drug development because of its role in an essential bacterial process and the lack of a homologous function in humans (Silverman, 1988 ▶). Genetic studies have established that bacteria carry either one or two alanine racemase genes (*alr* and/or *dadX*). The *alr* gene encodes the constitutively expressed biosynthetic enzyme that provides d-alanine necessary for peptidoglycan synthesis (Wasserman *et al.*, 1983 ▶). The catabolic *dadX* gene, which is present mainly in Gram-negative bacteria, encodes a second alanine racemase isozyme with expression induced by high levels of l-alanine in the medium (Strych *et al.*, 2000 ▶). The *dadX* gene product converts the abundant l-alanine to d-alanine, which is subsequently metabolized to pyruvate and used as an alternate carbon and energy source (Wasserman *et al.*, 1983 ▶; Wild *et al.*, 1985 ▶). Only one *alr* gene has been identified in the *C. difficile* genome, and as in other bacteria with only one *alr* gene this gene is likely to be essential for *C. difficile* growth (Monot *et al.*, 2011 ▶).

Structural studies of Alrs from bacteria such as *Geobacillus stearothermophilus* (Shaw *et al.*, 1997 ▶), *Streptomyces levendulae* (Noda *et al.*, 2004 ▶), *Pseudomonas aeruginosa* (LeMagueres *et al.*, 2003 ▶), *Mycobacterium tuberculosis* (LeMagueres *et al.*, 2005 ▶), *Escherichia coli* (Wu *et al.*, 2008 ▶), *Bacillus anthracis* (Au *et al.*, 2008 ▶; Couñago *et al.*, 2009 ▶), *Streptococcus pneumoniae* (Im *et al.*, 2011 ▶) and *Staphylococcus aureus* (Scaletti *et al.*, 2012 ▶) indicate that Alr is a homodimeric enzyme formed by head-to-tail association of two monomers. Each monomer consists of two domains: an eight-stranded α/β barrel at the N-terminus and β-strands at the C-terminus. The dimer contains two active sites, which are formed by the association of the N-terminal domain of one monomer and the C-terminal domain of the other. In each active site, the PLP cofactor resides in the mouth of the α/β barrel, forming a Shiff base with a catalytic lysine. Structures of Alr in complex with substrate analogs (Fenn *et al.*, 2003 ▶; Morollo *et al.*, 1999 ▶; Stamper *et al.*, 1998 ▶; Watanabe *et al.*, 2002 ▶) and site-directed mutagenesis studies of the enzyme (Watanabe *et al.*, 2002 ▶; Watanabe, Yoshimura *et al.*, 1999 ▶; Watanabe, Kurokawa *et al.*, 1999 ▶; Sun & Toney, 1999 ▶) have elucidated the reaction mechanism and verified the key roles of active-site residues. In general, Alr utilizes a stepwise two-base mechanism whereby two highly conserved lysine and tyrosine residues at the active site act as acid/base catalysts, assuming the roles of either donating or abstracting an α-hydrogen from the substrate. Structures of Alr with inhibitors, such as alanine phosphonate and d-cycloserine, show that these inhibitors covalently bind to the PLP cofactor (Fenn *et al.*, 2003 ▶).

The availability of structural information on Alrs from a range of bacterial species is crucial for the design of inhibitors that can serve as antibiotics. Although numerous structures of Alrs from aerobic and facultative anaerobes are available, very little is known to date about the homologous enzyme from the obligate anaerobe *C. difficile*. Thus, the successful purification, crystallization, structure determination and kinetic characterization of *C. difficile* alanine racemase (CdAlr) from the hypervirulent and multidrug-resistant strain 630 are presented here.

## 2. Materials and methods   

### 2.1. Expression and purification   

The amino-acid sequence of *C. difficile* strain 630 Alr (NCBI Reference Sequence YP_001089983.1) was used as a template for the chemical synthesis of DNA expression constructs encoding the 385-amino-acid protein as well as a point mutant carrying a lysine-to-threonine substitution at position 271, K271T (GenScript USA Inc., Piscataway, New Jersey, USA). These synthetic constructs were codon-optimized for expression in *E. coli* and cloned into the *Xba*I/*Bgl*II sites of pET-32a vector (Novagen, Madison, Wisconsin, USA). The expression plasmids were transformed into *E. coli* strain Rosetta 2 (pLysS) DE3 by heat shock. Positive colonies were identified by colony PCR with T7 promoter and T7 terminator primers (EMD Millipore, Rockland, Massachusetts, USA). Initial small-scale expression screens were performed on ten positive clones by culturing a single colony in 10 ml LB medium containing 50 µg ml^−1^ ampicillin to an OD_600_ of 0.7. Protein expression was induced by the addition of IPTG to a final concentration of 0.5 m*M* for 16 h at 288 K. The highest expressing clone was selected for large-scale expression. Cell pellets were harvested by centrifugation, suspended in 20 m*M* Tris pH 7.5 with protease inhibitors (Roche) and lysed using a French press. The lysate was clarified by centrifugation prior to Alr purification by anion-exchange chromatography (Q Sepharose Fast Flow, GE Healthcare) and elution with an NaCl gradient in lysis buffer. This was followed by further purification by size-exclusion chromatography (HiPrep 16/60 Sephacryl S-300 HR) with 100 m*M* Tris pH 7.5, 1 m*M* PLP, 1 m*M* TCEP. The typical yield of purified recombinant CdAlr was 15 mg from 1.2 l of culture. Coomassie-stained SDS gels were used to assess the purity of the recombinant proteins, which were dialyzed into storage buffer (50 m*M* Tris pH 7.5, 10 m*M* PLP, 1 m*M* DTT) and stored at −80°C until use.

### 2.2. Enzyme kinetics   

The rate of conversion of l-alanine to d-alanine was determined by measuring the amounts of both enantiomers by HPLC as described previously (Lee *et al.*, 2013 ▶). The kinetics of alanine racemization in both the forward (l-alanine to d-alanine) and the reverse (d-alanine to l-alanine) directions were determined for recombinant wild-type (CdAlr_WT_) and K271T mutant (CdAlr_K271T_) enzymes. The reaction mixtures consisted of l-alanine or d-alanine at a range of concentrations (0.2, 0.39, 0.78, 1.56, 3.12, 6.25, 12.5, 25 and 50 m*M*) in 50 m*M* sodium borate pH 8.5 buffer. The reaction was initiated by the addition of either CdAlr_WT_ (480 ng) or CdAlr_K271T_ (6 ng). The amount of each component and the reaction times were determined *via* optimization studies. As the reaction proceeded, aliquots were removed at 0.5 min intervals over a total of 4 min and the reaction was quenched with four volumes of methanol. The l-alanine and d-alanine enantiomers in the methanol extracts were separated based on their differential diastereomer stability *via* isocratic HPLC (Agilent 1100 Chemstation) using a Nucleosil Chiral-1 column (Machery-Nagel, Germany) with a mobile phase containing 0.5 m*M* copper sulfate solution at a flow rate of 0.3 ml min^−1^ at 333 K. The percentage of d-alanine product in the total alanine mixture was calculated from the peak areas in the HPLC profile at *A*_240 nm_. The rates of production of l-alanine or d-alanine (m*M* min^−1^) were plotted against the substrate concentration and the slopes were used in the Michaelis–Menten analysis to obtain *V*_max_ and *K*_m_ (*GraphPad Prism* 6, GraphPad Software, La Jolla, California, USA). The *k*_cat_ was calculated from the *V*_max_ [*k*_cat_ = *V*_max_ (m*M* min^−1^)/Alr concentration (m*M*)]. The results shown are the mean ± SD of three independent experiments.

### 2.3. Complementation analysis   

For complementation analysis, two plasmids in which the *alr* genes were constitutively expressed from the *lac* promoter were constructed by inserting synthetic genes encoding *C. difficile* wild-type Alr and K271T Alr into the *Xba*I–*Sac*I sites of the pUC57 vector. The *alr*/*dadX* double-knockout *E. coli* strain MB2159 (Strych *et al.*, 2001 ▶) was transformed with the plasmids by electroporation (Bio-Rad; 25 µF, 200 Ω and 1.65 kV). Transformants were selected on LB medium in the absence of exogenous d-alanine.

### 2.4. Size-exclusion chromatography and multi-angle light scattering (SEC–MALS)   

SEC–MALS experiments involved loading 10 µg of each protein sample onto a stationary phase comprised of a TSKgel Super SW mAb HTP column (Tosoh Biosciences, King of Prussia, Pennsylvania, USA) at a flow rate of 0.25 ml min^−1^ with an Agilent 1260 Infinity series HPLC. The mobile phase buffer was phosphate-buffered saline pH 7.4. Each experiment was performed in triplicate. The detector system consisted of a UV detector (Agilent) and a miniDAWN triple-angle light-scattering detector (Wyatt Technology) with an Optilab rEX differential refractometer (Wyatt Technology) connected in series. The refractometer provides a continuous index of protein concentration using a d*n*/d*c* (refractive-index increment) value of 0.185 ml mg^−1^. Bovine serum albumin was used as an isotropic scatterer for detector normalization. Since the light scattered by a protein is directly proportional to its weight-average molecular mass and concentration, molecular masses were calculated from the light-scattering and interferometric refractometer data using the *ASTRA* 6.0 software.

### 2.5. Crystallization   

Both the purified recombinant wild-type (CdAlr_WT_) and K271T mutant (CdAlr_K271T_) protein samples were concentrated to 32 mg ml^−1^ in buffer composed of 10 m*M* PLP and 1 m*M* TCEP in 50 m*M* Tris–HCl pH 8 prior to setting up crystal screens. Initial crystallization screens were carried out on samples of both proteins using commercial sparse-matrix screens from Hampton Research and Qiagen, including The Classics I, Classics II, Cryos, PEGs and AmSO_4_ Suites and Index at both 293 and 298 K by vapor diffusion in sitting drops. Drops were prepared by mixing 2 µl protein solution with 1.5 µl reservoir solution. Large yellow plate-like crystals of both enzymes were obtained using high concentrations of polyethylene glycol (PEG) at both temperatures. These crystals were typically 0.4 × 0.5 × 0.1 mm in size. The CdAlr_WT_ crystals were obtained in 0.2 *M* ammonium sulfate, 0.1 *M* bis-tris pH 6.5, 25%(*w*/*v*) PEG 3350. Virtually identical crystals were obtained at different pH and buffer conditions (Tris–HCl pH 8.5, dl-malic acid pH 7.0, HEPES pH 7.5 or bis-tris pH 5.5) or using different salts (magnesium chloride, sodium formate, lithium sulfate, sodium chloride or potassium bromide). The largest crystals of CdAlr_K271T_ were obtained from 0.17 *M* lithium sulfate, 0.085 *M* Tris–HCl pH 8.5, 25.5%(*w*/*v*) PEG 4000, 20%(*v*/*v*) glycerol. The CdAlr co-crystals with cycloserine (CdAlr_cycloserine_) were obtained in 100 m*M* cycloserine, 200 m*M* sodium formate, 20%(*w*/*v*) PEG 3350. Prior to data collection, all crystals that were grown in crystallization solutions with less than 15%(*v*/*v*) glycerol were incubated for approximately 30 s in cryoprotecting buffers comprised of the crystallization solution with 15%(*v*/*v*) glycerol.

### 2.6. Data collection and structure determination   

X-ray diffraction data sets were collected at the Baylor College of Medicine core facility using a Rigaku HTC detector and a Rigaku FR-E+ SuperBright microfocus rotating-anode generator with VariMax HF optics. Each data set was collected at 100 K from a single crystal with exposure times of 45 s for 0.5° oscillations using the *CrystalClear* (d*TREK) package (Pflugrath, 1999 ▶). The data were processed using *iMosflm* (Leslie, 2006 ▶; Battye *et al.*, 2011 ▶). For structure determination, data were collected from three crystals. The first data set was for the complex of the wild-type enzyme with the PLP cofactor (CdAlr_PLP_). The second data set was for a co-crystal with the inhibitor cycloserine (CdAlr_cycloserine_), while the third was for the K271T mutant enzyme in complex with PLP (CdAlr_K271T_). Both the CdAlr_PLP_ and the CdAlr_K271T_ crystals belonged to the monoclinic space group *P*2_1_. The former has unit-cell parameters *a* = 85.2, *b* = 93.3, *c* = 107 Å, α = γ = 90, β = 91°, while the latter has unit-cell parameters *a* = 49.97, *b* = 154.3, *c* = 55.8 Å, α = γ = 90, β = 113°. The CdAlr_cycloserine_ crystal belonged to the orthorhombic space group *P*2_1_2_1_2_1_, with unit-cell parameters *a* = 57.9, *b* = 139.1, *c* = 144.9 Å, α = β = γ = 90°. Despite having visible reflections to 2.26 Å resolution, the CdAlr_cycloserine_ data were less than 60% complete at high resolution. Attempts to collect more complete data just increased the redundancy of the data without increasing the completeness owing to the marked diffraction anisotropy of the crystal. According to Wlodawer and coworkers, marked anisotropy of diffraction occurs when the reflection intensity in one direction extends to higher resolution than in the other directions; since contemporary refinement programs can effectively handle the refinement of this type of data, the best remedy in this situation is to use all the weak high-resolution data, *i.e.* the maximum possible number of reflections (Wlodawer *et al.*, 2013 ▶). Setting an arbitrary cutoff to maintain an overall completeness of greater than 80% results in a significantly less informative 3.4 Å resolution structure. Additionally, the overall *R*_merge_ of the three data sets are artificially inflated by the high multiplicity of the data sets, since multiplicity and *R*_merge_ are interdependent variables (Wlodawer *et al.*, 2013 ▶).

All structures were solved by molecular replacement with *Phaser* (McCoy *et al.*, 2005 ▶; Storoni *et al.*, 2004 ▶). The molecular-replacement search model was a monomer of alanine racemase from *S. aureus* (PDB entry 4a3q) stripped of all waters and ligands (Scaletti *et al.*, 2012 ▶). Molecular replacement was followed by iterative cycles of model building with *Coot* (Emsley *et al.*, 2010 ▶) and structure refinement with *REFMAC*5 (Murshudov *et al.*, 2011 ▶) within the *CCP*4 package (Winn *et al.*, 2011 ▶). In the initial refinement stages, noncrystallographic symmetry (NCS) restraints were utilized. Additionally, the refined coordinates were subjected to *PDB-REDO* (Joosten, Salzemann *et al.*, 2009 ▶; Joosten, Womack *et al.*, 2009 ▶; Joosten *et al.*, 2012 ▶) to optimize the model, followed by several additional cycles of manual building to fix out-of-register errors and any other badly fitting regions that were introduced after *PDB-REDO*. Data-collection and refinement statistics are shown in Table 1 ▶. The atomic coordinates and structure factors have been deposited in the Protein Data Bank as entries 4lus (CdAlr_PLP_), 4luy (CdAlr_K271T_) and 4lut (CdAlr_cycloserine_).

## 3. Results and discussion   

### 3.1. Recombinant CdAlr is functionally active   

**Figure 1:**
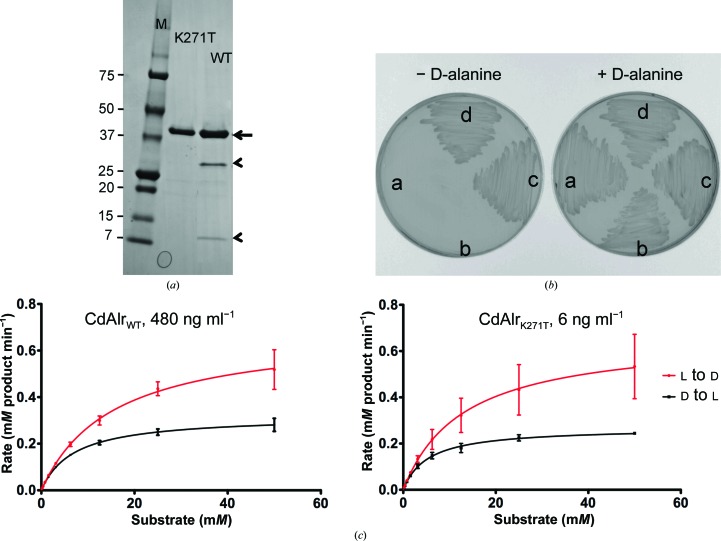
Biological and enzymatic activity of recombinant CdAlr. (*a*) Coomassie Blue-stained reduced SDS–PAGE gel of recombinant CdAlr_WT_ and CdAlr_K271T_ following anion-exchange and size-exclusion chromatography. The arrow indicates the full-length molecular weight 43 303 bands corresponding to monomeric protein. The arrowheads indicate the cleavage products in CdAlr_WT_ and lane *M* contains molecular-weight markers (labeled in kDa). (*b*) Growth rescue of a d-alanine auxotroph by plasmids producing recombinant CdAlr_WT_ and CdAlr_K271T_. Growth or lack thereof of *E. coli* MB2159 (labeled a) and of *E. coli* MB2159 transformed with pUC57 vector (labeled b), wild-type *C. difficile alr* (labeled c) and *C. difficile* K271T mutant *alr* (labeled d) on LB medium supplemented with 50 µg ml^−1^ d-alanine (+ d-alanine) and LB medium without supplementation (− d-alanine). (*c*) Enzyme activity plots of purified recombinant CdAlr_WT_ and CdAlr_K271T_.

Analysis of purified recombinant CdAlr_WT_ by Coomassie-stained SDS gels and subsequent mass spectrometry revealed a major species corresponding to the full-length protein (molecular weight 43 306) and two minor species of molecular weights 30 407 and 12 917 (Fig. 1 ▶ *a*). Both minor species interact tightly with the full-length protein and cannot be separated by additional size-exclusion chromatography. Subsequent peptide sequencing revealed that the minor species corresponded to amino acids 1–271 and 272–382 and that the two peptides resulted from a cleavage of unknown nature at position 271. To prevent this cleavage, the lysine at position 271 was mutated to a threonine: K271T. Thr was chosen because homologous Alrs from *S. aureus*, *M. tuberculosis*, *B. anthracis* and *G. stearothermophilus* that are readily purified and crystallized have a Thr at position 271. The K271T single substitution enabled the production and purification of homogeneous full-length CdAlr protein as assessed by SDS–PAGE (Fig. 1 ▶ *a*) and mass spectrometry (data not shown).

The biological activity and functionality of the synthetic constructs were determined by evaluating their ability to complement an *E. coli* d-alanine auxotroph. When transformed into an *E. coli alr*/*dadX* mutant, the plasmids encoding the wild-type and K271T mutant CdAlr proteins were able to complement the d-alanine auxotrophy and restore its growth in d-alanine-deficient medium (Fig. 1 ▶ *b*), demonstrating that both recombinant proteins are biologically active.

After confirmation of their biological activity, the racemase activity of recombinant wild-type (CdAlr_WT_) and K271T mutant (CdAlr_K271T_) enzymes were assessed by testing their ability to catalyze the interconversion of l-alanine and d-alanine. Both recombinant enzymes were catalytically active (Fig. 1 ▶ *c*). Purified CdAlr_WT_ catalyzed the interconversion of l-alanine to d-alanine *in vitro*, with a *K*_m_ of 17.6 m*M* for l-alanine and a *V*_max_ of 82.1 units mg^−1^ in the forward reaction and a *K*_m_ of 7 m*M* for d-alanine and a *V*_max_ of 26.7 units mg^−1^ in the reverse reaction. CdAlr_K271T_ had a *K*_m_ of 13.8 m*M* for l-alanine and a *V*_max_ of 4533 units mg^−1^ in the forward reaction and a *K*_m_ of 5.4 m*M* for d-alanine and a *V*_max_ of 1797 units mg^−1^ in the reverse reaction. CdAlr_K271T_ had a significantly higher catalytic activity than CdAlr_WT_ (Table 2 ▶). The lysine-to-threonine substitution is relatively conservative in that both are polar and hydrophobic residues, thus the observation that the smaller (molecular mass 114 *versus* 146) uncharged amino acid threonine has such a large impact on the catalytic activity of CdAlr is somewhat puzzling. As mentioned previously, the main difference between the recombinant CdAlr_WT_ and CdAlr_K271T_ protein samples is the presence of two fragments in the former (Fig. 1 ▶ *a*). The fragments are likely to be enzymatically inactive since the head-to-tail association of two monomers is necessary to form the dimeric active sites of Alr, in which both the N-terminal and C-terminal residues are essential for catalytic activity. Additionally, these inactive species are likely to associate with full-length monomers to form inactive or partially active dimers, which could explain the inability to separate the cleaved species by size-exclusion chromatography as well as the apparent monodisperity of the sample *via* SEC–MALS. Therefore, the CdAlr_WT_ preparation is likely to contain a heterogeneous population of dimers made up of both full-length and truncated monomers, which could account for the need to use substantially more CdAlr_WT_ than CdAlr_K271T_ in the enzymatic assays as well as the lower catalytic activity.

The catalytic activities of CdAlr_WT_ and CdAlr_K271T_ were compared with those of several Alr homologs that have been reported by others. As summarized in Table 2 ▶, CdAlr_K271T_ appears to be significantly more active than CdAlr_WT_ and the Alrs from *G. stearothermophilus*, *S. lavendulae*, *M. tuberculosis* and *M. smegmatis*, as reflected by a 50-fold to 1000-fold higher *k*_cat_ (Table 2 ▶). Significant differences in catalytic activities among Alrs with high sequence identities have been reported (Strych *et al.*, 2000 ▶). We previously reported that the Alr of *M. smegmatis*, which shares 65% sequence identity with its homolog from *M. tuberculosis*, is 20-fold more active (Lee *et al.*, 2013 ▶). The molecular basis for the variations, although still remaining elusive, has been attributed to differences in the monomer–dimer equilibrium (Ju *et al.*, 2011 ▶). The more active isoforms are thought to exist predominantly as catalytically active dimers owing to the increased propensity of the monomers to associate, while the less active isoforms, with their lower monomer association constants, are largely monomeric. Accordingly, the high catalytic activity of CdAlr_K271T_ could arise from the formation of a tight dimer that hardly dissociates. The biological significance of Lys271 in CdAlr instead of the Thr commonly found at this position in Alr homologs, including those from *S. aureus*, *M. tuberculosis*, *B. anthracis* and *G. stearothermophilus*, is not known. The confusion is further compounded by the observation that the *S. lavendulae* homolog (PDB entry 1vft; Noda *et al.*, 2004 ▶), which has a similar *k*_cat_ as CdAlr, has a His in the corresponding position. A more detailed analysis of the monomer association constants among the various Alrs is needed to shed light on the differences in the catalytic activities. It is also unclear whether the native Alr from the bacterium *C. difficile* undergoes cleavage similar to that observed for the recombinant enzyme produced in *E. coli*; this is worth exploring in order to determine whether *C. difficile* uses this mechanism to modulate Alr activity during anaerobic growth.

### 3.2. Recombinant CdAlr is a dimer in solution   

**Figure 2:**
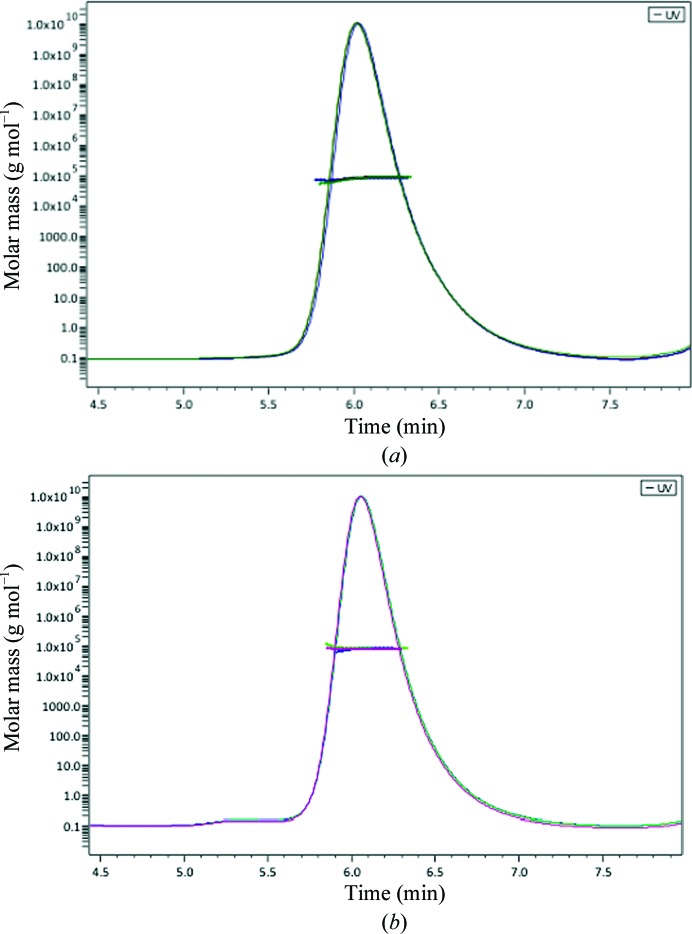
Representative SEC–MALS elution profiles for (*a*) CdAlr and (*b*) CdAlr_K271T_. Molecular masses for three runs are plotted against elution time for (*a*) CdAlr_PLP_ and (*b*) CdAlr_K271T_.

In order to determine the oligomeric form of the protein in solution, the absolute molecular masses of both CdAlr_WT_ and CdAlr_K271T_ were measured by size-exclusion chromatography and multi-angle light scattering (SEC–MALS). Both proteins gave single monodisperse peaks on the sizing column (Fig. 2 ▶). The molecular masses were determined to be 81.09 ± 2.04 and 80.53 ± 1.98 kDa for CdAlr_WT_ and CdAlr_K271T_, respectively, indicating that both proteins form dimers in solution. These values are slightly smaller than the theoretical molecular weights of 86.6 and 86.4 kDa for the dimers of CdAlr_WT_ and CdAlr_K271T_, respectively. The differences in mass are comparable to the dynamic light-scattering studies of the homolog from *S. aureus*, which revealed a dimer with 98.6% of the theoretical molecular mass (Scaletti *et al.*, 2012 ▶).

### 3.3. Overall structure of CdAlr   

Three crystal structures of CdAlr are reported in this study. The first is the structure of the enzyme in complex with the PLP cofactor (CdAlr_PLP_). The second structure is that of the co-crystal with the inhibitor cycloserine (CdAlr_cycloserine_). The third structure is that of the K271T mutant enzyme in complex with PLP (CdAlr_K271T_). Uniquely, the CdAlr_PLP_ crystal structure is a tetramer, while the other two structures are dimers. The CdAlr_PLP_ structure gives the highest resolution data, but it also has the highest level of disorder and lacks several residues in proximity to the binding cavity and the dimer interface. Key disordered regions in monomer *A* include all residues from Lys258 to Thr279; in monomer *B* the disordered residues are Ser133–Gly139 and Glu173–Glu177, while in monomer *D* residues Gly197–Ile200 are disordered. Some of these residues were disordered in other Alr structures (Au *et al.*, 2008 ▶; Couñago *et al.*, 2009 ▶; Im *et al.*, 2011 ▶; Scaletti *et al.*, 2012 ▶). The CdAlr_PLP_ tetramer is a dimer of two almost identical dimers, with an r.m.s.d. of 0.274 Å for the superpositioning of main-chain atoms of the dimers. Each dimer is structurally similar to the homodimers of CdAlr_K271T_ (r.m.s.d. of 0.288 Å to one dimer and 0.361 Å to the other) and CdAlr_cycloserine_ (r.m.s.d. of 0.397 Å to one dimer and 0.511 Å to the other). The structure of CdAlr_K271T_ is the most complete structure and each monomer has ordered main-chain and side-chain electron-density maps for 383 of 385 amino acids with the two disordered residues are at the N-terminus.

**Figure 3:**
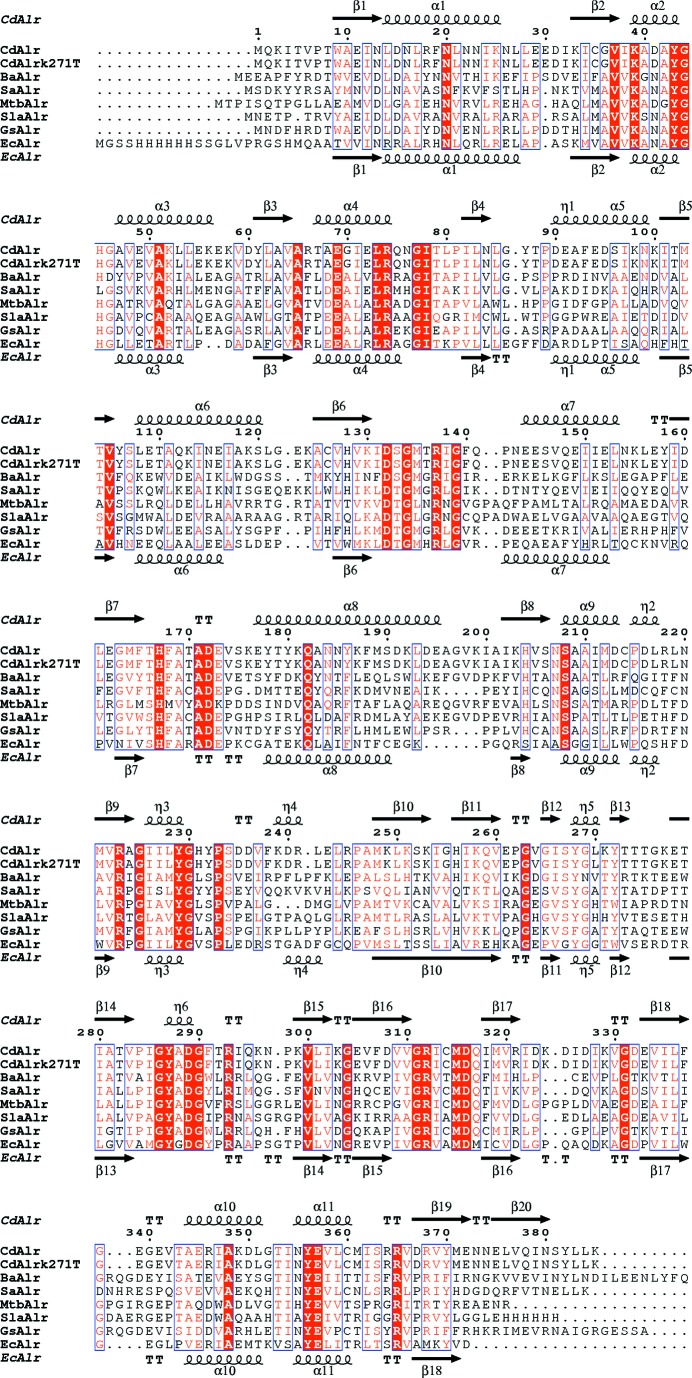
Comparison of CdAlr with selected representative Alrs. The amino-acid sequence alignment of representative Alrs showing structural elements of CdAlr and EcAlr was generated with *ESPript*3.0 (Gouet *et al.*, 2003 ▶). Secondary-structure elements are as follows: α-helices are shown as large coils labeled α, 3_10_-helices are shown as small coils labeled η, β-strands are shown as arrows labeled β and β-turns are labeled TT. Identical residues are shown on a red background, conserved residues are shown in red and conserved regions are shown in blue boxes. The representative Alrs are the homologs from *G. stearothermophilus* (GsAlr; PDB entry 1xqk; Fenn *et al.*, 2005 ▶), *B. anthracis* (BaAlr; PDB entry 3ha1; Couñago *et al.*, 2009 ▶), *S. lavendulae* (SlaAlr; PDB entry 1vft; Noda *et al.*, 2004 ▶), *S. aureus* (SaAlr; PDB entry 4a3q; Scaletti *et al.*, 2012 ▶), *M. tuberculosis* (MtbAlr; PDB entry 1xfc; LeMagueres *et al.*, 2005 ▶) and *E. coli* (EcAlr; PDB entry 2rjg; Wu *et al.*, 2008 ▶).

**Figure 4:**
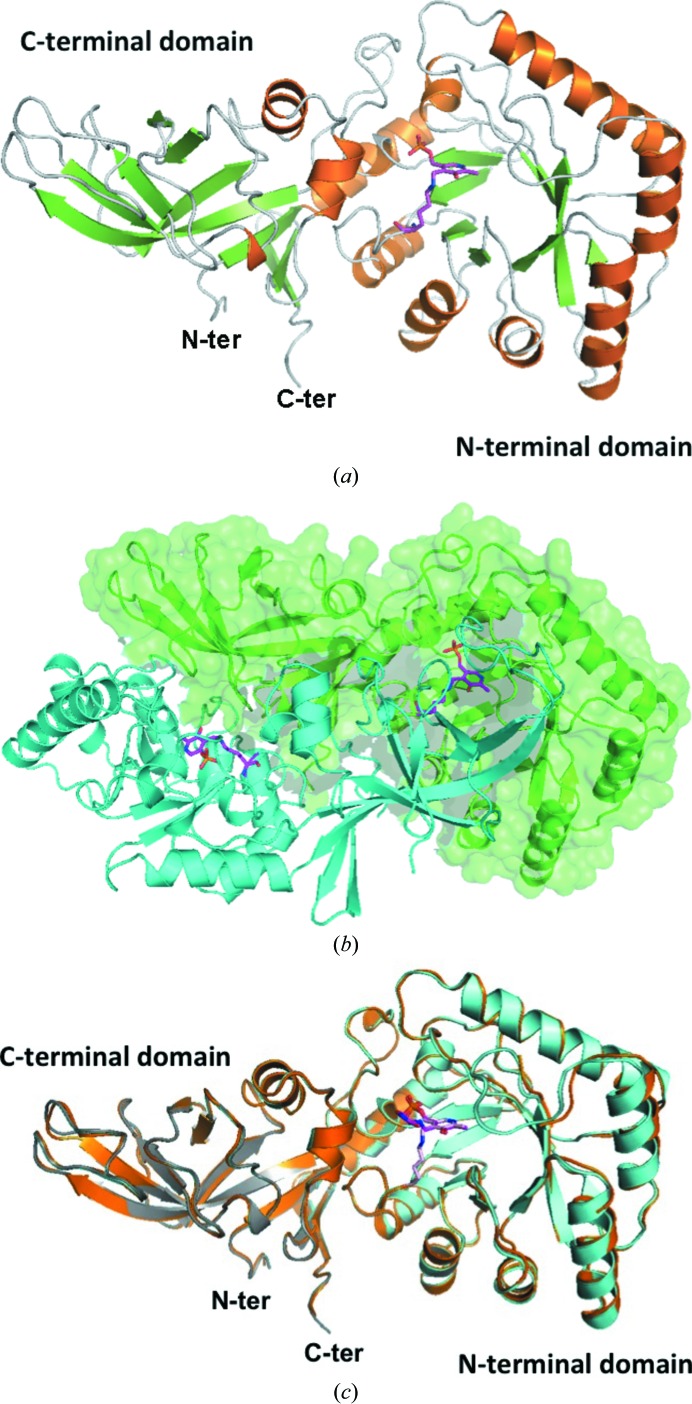
Structure of *C. difficile* alanine racemase. (*a*) Monomer of *C. difficile* alanine racemase. Ribbon diagram with α-helices in orange and β-strands in green; the PLP cofactor bound to Lys39 is shown as magenta sticks. (*b*) Dimer of CdAlr_K271T_. One monomer is shown in blue ribbon and the second is depicted as a green ribbon and surface. The PLP adduct, Llp39, is shown as magenta sticks. (*c*) Superposed ribbon structures of monomers of CdAlr_PLP_ (blue), CdAlr_K271T_ (grey) and CdAlr_cycloserine_ (orange). Llp39 is shown as magenta sticks and DCS is shown as blue sticks.

The topology of CdAlr_K271T_ was chosen to describe the overall enzyme topology since it is the most complete structure. The secondary structure of each monomer is approximately 25% strands, 26% α-helices, 5% 3_10_-helices and 44% loop and turns. There are five α/β-motifs, four β-hairpins, four β-bulges, four strands, 11 α-helices, six 3_10_-helices, 13 helix–helix interactions, 25 β-turns and four γ-turns (Fig. 3 ▶). Each monomer is made up of two domains, an N-terminal eight-stranded α/β-barrel PLP-binding domain and a C-terminal domain, linked by a short hinge region (Fig. 4 ▶ *a*). The homodimer is the minimal functional unit of CdAlr since each active site is formed at the interface of the N-terminal domain of one monomer and the C-terminal domain of the second monomer. The two monomers form a tight interface across the twofold axis of symmetry (Fig. 4 ▶ *b*). The interacting interface between the monomers is 3387 Å^2^ (66 amino-acid residues) from one monomer. The interface is mediated by 31 hydrogen bonds and corresponds to 19% of the surface area, which is comparable to other Alrs (Table 3 ▶ *b*).

### 3.4. Active site of CdAlr   

**Figure 5:**
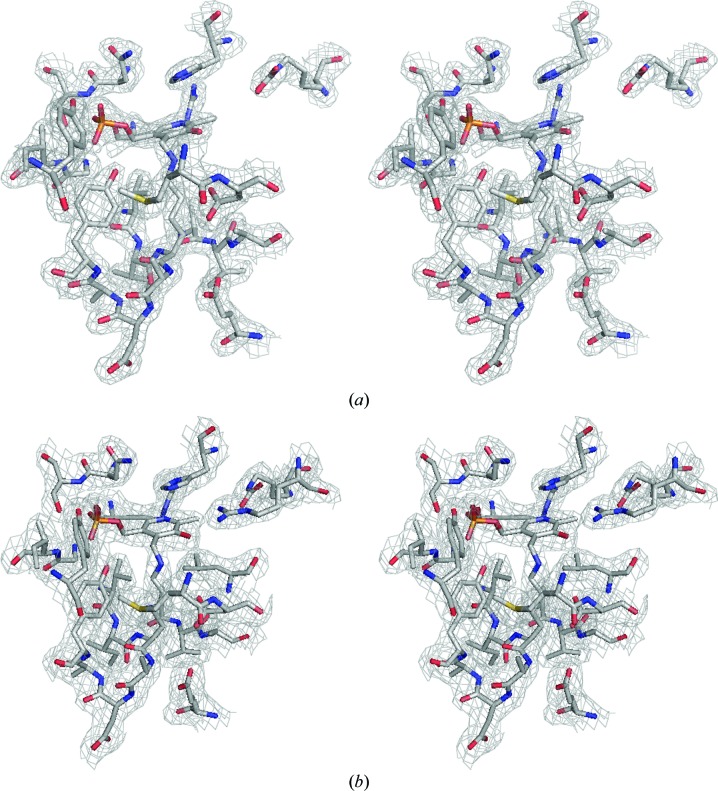

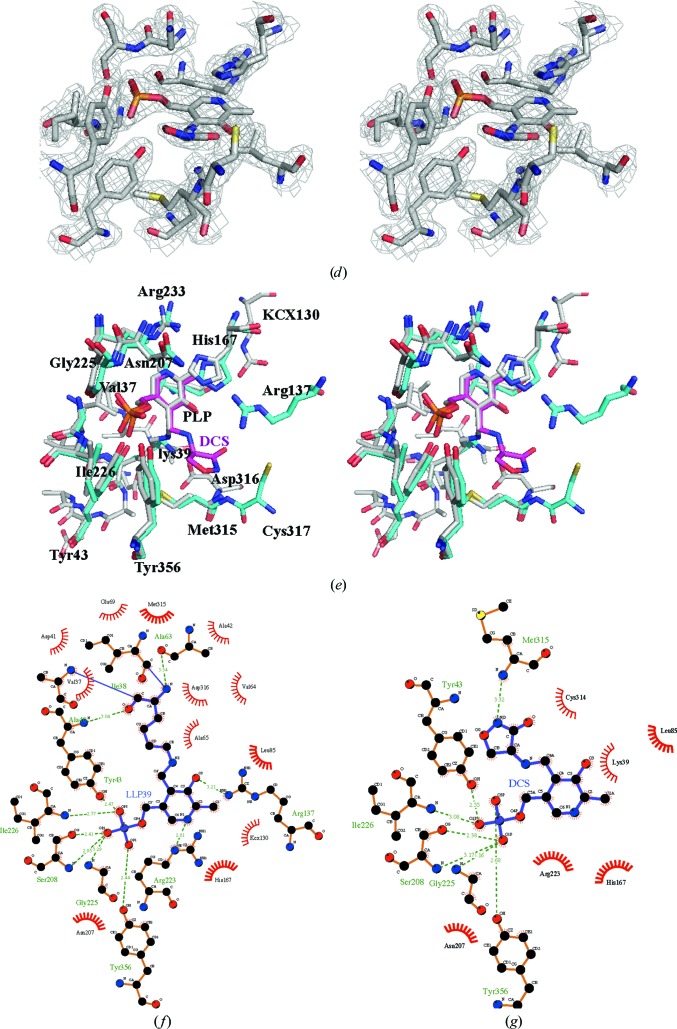
The Alr active site. Stereoview of cofactor PLP and active-site residues in a 2*F*_o_ − *F*_c_ electron-density map contoured at 1.2σ for (*a*) CdAlr_PLP_ (PDB entry 4lus) and (*b*) CdAlr_K271T_ (PDB entry 4luy). (*c*) The fit of active-site residues in a 2*F*_o_ − *F*_c_ electron-density map contoured at 2σ for CdAlr_cycloserine_ (PDB entry 4lut). (*d*) A stereoview of superposed residues in the active site of CdAlr_K271T_ (gray) and CdAlr_cycloserine_ (green). DCS is the covalent adduct of cycloserine and PLP, shown in magenta, and KCX is *N*-6-carboxyl-l-lysine. (*e*, *f*) Schematic plots of the interactions in the active sites of (*e*) CdAlr_K271T_ and (*f*) CdAlr_cycloserine_. The interaction figures were generated with *LigPlot*+ v.1.4.5 (Laskowski & Swindells, 2011 ▶).

All three CdAlr structures contain a ligand in the active site, and there is clear unambiguous density for the ligands in all cases (Figs. 5 ▶ *a*, 5 ▶ *b* and 5 ▶ *c*). Two structures (CdAlr_K271T_ and CdAlr_PLP_) contain the cofactor PLP, while one (CdAlr_cycloserine_) contains the covalent adduct of the inhibitor cycloserine and the PLP cofactor. In each active site of CdAlr_K271T_ and CdAlr_PLP_, the PLP cofactor resides in the mouth of the α/β-barrel, forming a covalent adduct with Lys39 *via* an internal aldimine linkage, forming 2-lysine-(3-hydroxy-2-methyl-5-phosphono­oxy­methyl-pyridin-4-ylmethane), Llp (Figs. 5 ▶ *a* and 5 ▶ *b*). Covalent bonding to the residue corresponding to Lys39 was observed in other structures of alanine racemase in complex with PLP or substrate analogues (Fenn *et al.*, 2003 ▶; Morollo *et al.*, 1999 ▶; Stamper *et al.*, 1998 ▶; Watanabe *et al.*, 2002 ▶). The covalent adduct Llp39 forms a network of hydrogen bonds with several amino-acid residues, including Arg223, Ser208, Tyr356, Gly225, Ile226, Tyr43, Arg137 and KCX130, which is carbamylated Lys130 (Fig. 5 ▶ *e*).

Despite conformational differences proximal to the active site, these key residues in the active site of CdAlr are well conserved in other Alrs (Watanabe *et al.*, 2002 ▶; Watanabe, Yoshimura *et al.*, 1999 ▶; Watanabe, Kurokawa *et al.*, 1999 ▶; Sun & Toney, 1999 ▶), suggesting similar reaction mechanisms. In general, Alrs use a stepwise two-base mechanism whereby two highly conserved active-site residues, lysine (Lys39) and tyrosine (Tyr268) act as acid/base catalysts, either donating or abstracting an α-H atom from the substrate (Watanabe *et al.*, 2002 ▶). In the CdAlr_cycloserine_ structure, cycloserine forms a covalent adduct with PLP, d-pyridoxyl-*N*,*O*-cycloserylamide-5-monophosphate (DCS). The formation of the covalent adduct prevents the covalent interaction with Lys39 that was observed in the CdAlr_PLP_ structure (Fig. 5 ▶). Several hydrogen bonds are retained in the CdAlr_cycloserine_ and CdAlr_K271T_ (CdAlr_PLP_) structures, notably those between Tyr43, Ile226, Gly225 and Tyr356. An additional hydrogen bond is formed between Tyr268 and DCS that is not present in the CdAlr_PLP_ and CdAlr_K271T_ structures (Figs. 5*e* and 5*f*).

**Figure 6:**
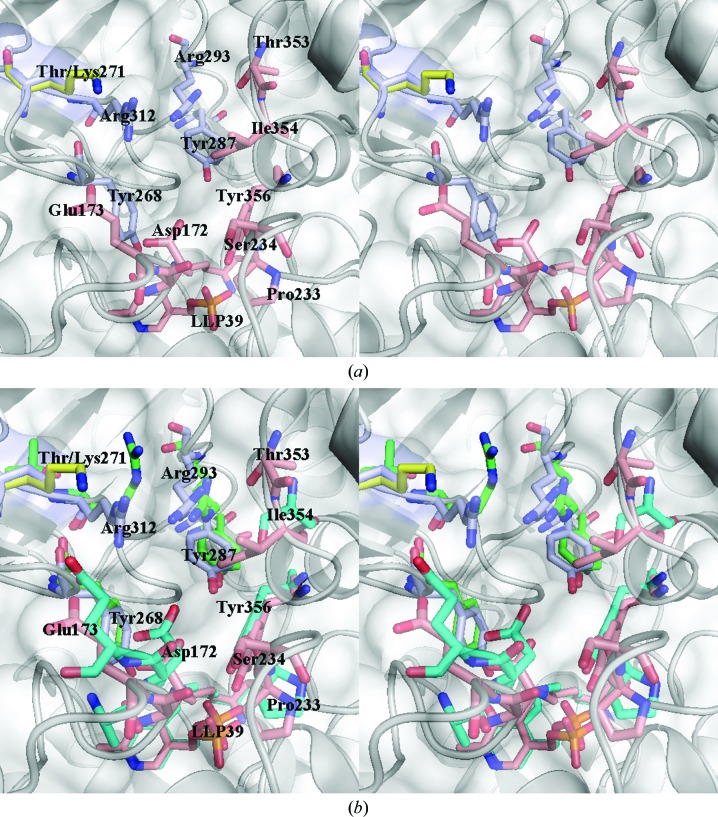
Substrate entryway. (*a*) A stereoview of the substrate entryway of CdAlr_K271T_ superposed with CdAlr_cycloserine_. (*b*) The same stereoview of the substrate entryway of CdAlr_K271T_ superposed with that of EcAlr. In both figures residues from one monomer of CdAlr_K271T_ are shown in pink, while residues from the second monomer are shown in blue and Lys271 from CdAlr_WT_ is shown in yellow. Corresponding residues in EcAlr are colored green and aquamarine, respectively. The transparent surface and ribbon diagram shown are of CdAlr_K271T._

Leading to the active site is the substrate entryway, which is comprised of conserved residues that have been considered to be potential targets for drug design (LeMagueres *et al.*, 2005 ▶; Wu *et al.*, 2008 ▶). As was the case in other Alrs, the residues lining the substrate entryway are from two monomers, and include residues from across the dimer interface (Fig. 6 ▶ *a*). Two monomers contribute to the substrate entryway. One monomer contributes the key residues Asp172, Tyr356, Thr353, Ser234, Pro233 and Ile354, while another monomer across the dimer interface contributes Lys/Thr271, Arg312, Arg293 and Tyr287 to the entryway (Fig. 6 ▶ *a*). Lys271 lies on a loop in the substrate entryway of the active site and it has previously been shown that mutations in this region affect the activity of EcAlr (Wu *et al.*, 2008 ▶). Interestingly, the residues in the substrate entryway of EcAlr superpose quite well with those of CdAlr and are well conserved (Fig. 6 ▶ *b*). The K271T mutation results in a more open and accessible entryway (Fig. 6 ▶ *a*), which may contribute to the observed increased activity.

### 3.5. Comparison with other alanine racemase structures   

The structures that were most similar to CdAlr were identified using the Structure Similarity option in *PDBeFold* (http://www.ebi.ac.uk/msd-srv/ssm/). The criteria for identifying similar structures included the r.m.s.d. for alignment of all main-chain atoms, alignment length and number of gaps. Although it only has 37% sequence identity to CdAlr, the crystal structure of the *G. stearothermophilus* (GsAlr) Y265F mutant alanine racemase in complex with cycloserine (PDB entry 1xqk; Fenn *et al.*, 2005 ▶) has the greatest overall three-dimensional structural alignment with the CdAlr structures. Other complexes and mutants of GsAlr also ranked high in terms of structural similarity. The corresponding PDB entries are 1l6g (Watanabe *et al.*, 2002 ▶), 1epv (Fenn *et al.*, 2003 ▶) and 2sfp (Fenn *et al.*, 2003 ▶). Other proteins that share significant structural similarity include the *B. anthracis* homolog (PDB entry 3ha1; Couñago *et al.*, 2009 ▶) as well as the *S. aureus* homologue (PDB entry 4a3q; Scaletti *et al.*, 2012 ▶). These Alrs share ∼37% sequence identity with CdAlr. The monomers of all these structures superpose well and have r.m.s.d.s for alignment of all main-chain atoms varying from 1.45 to 1.62 Å against the three CdAlr structures, despite the observation that CdAlr has longer helices, strands and loops than many of the other Alrs.

The N-terminal and C-terminal domains of all reported Alr structures are connected by a short hinge region, and it was previously revealed that the hinge angles between the N- and C-terminal of Alrs vary (LeMagueres *et al.*, 2003 ▶; Im *et al.*, 2011 ▶). To compare the hinge angles of CdAlr with those of representative Alrs, the angles were calculated using Peter Sun’s applet *Hinge* (http://exon.niaid.nih.gov/sis/hinge_file.html). The calculated hinge angle for CdAlr was 123.50°, which is comparable to those of BaAlr (123.34°) and GsAlr (123.22°). SlaAlr (124.76°) has a slightly larger hinge angle than CdAlr, while the hinge angles are larger for SaAlr (132.22°), MtbAlr (136.16°) and EcAlr (142.20°) (Table 3 ▶ *a*). There does not seem to be any correlation between the size of the hinge angle and the number of atoms in each domain. Additionally, there does not appear to be any correlation between hinge-angle size and catalytic activity (Tables 2 ▶ and 3 ▶ *a*).

CdAlr has a larger monomer surface area than many of the other Alrs, despite having a similar number of amino-acid residues (Table 3 ▶ *b*). The regions that are most different among all of the Alrs are in the N- and C-terminal loops as well as in the loop regions proximal to the dimer interface and the active site (Au *et al.*, 2008 ▶; Couñago *et al.*, 2009 ▶; Kanodia *et al.*, 2009 ▶; Im *et al.*, 2011 ▶; Scaletti *et al.*, 2012 ▶). These variations in the dimer interface contribute to differences in the buried surface area of the different Alrs (Table 3 ▶). The size of the dimeric buried surface area does not correlate to Alr enzymatic activity, as measured in terms of the ability to interconvert l-alanine to d-alanine (Tables 2 ▶ and 3 ▶ *b*).

### 3.6. Comparison of CdAlr with *E. coli* Alr   

**Figure 7:**
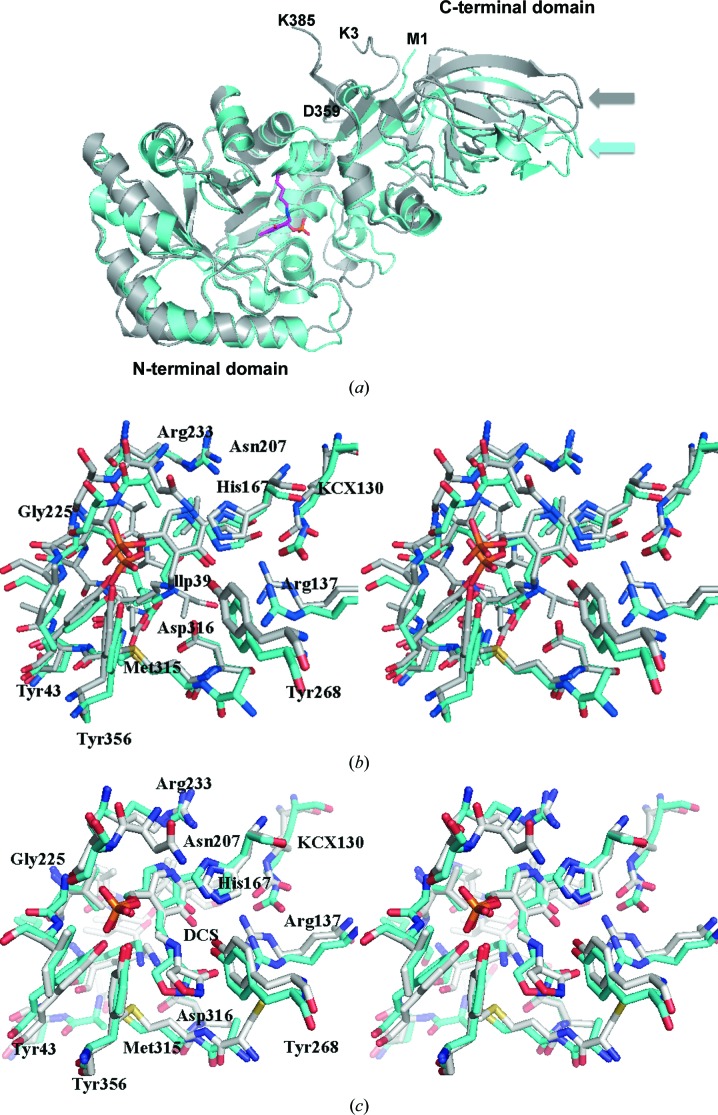
Comparison of *C. difficile* and *E. coli* Alr. (*a*) Superposed ribbon diagram of CdAlr_K271T_ (gray) with EcAlr (cyan). The larger hinge angle of EcAlr is obvious from the displacement of the corresponding loops in the C-terminal domain, indicated by arrows. The N-terminal residue (Lys3) and C-terminal (Lys385) residue of the CdAlr_K271T_ structure are labeled. Also labeled are the N-terminal (Met1) and C-terminal (Asp359) residues of the EcAlr structure. (*b*) A stereoview of the superposed active sites of CdAlr_K271T_ (gray) and EcAlr (green). (*c*) A stereoview of the superposed residues in the active site of CdAlr_cycloserine_ (gray) and EcAlr in complex with cycloserine (green).

In order to selectively target CdAlr for drug development, its differences from Alr from gut bacteria such as *E. coli* have to be identified. Structures of *E. coli* Alr (EcAlr) have been reported, including those of mutants as well as the complex with cycloserine (Wu *et al.*, 2008 ▶). The dimers of EcAlr and CdAlr are quite similar, having a main-chain r.m.s.d. of 1.9 Å for the cycloserine structures or the PLP structures. Interestingly, when the monomers are superposed based on the conserved N-terminal domain, the large difference in the hinge angle is evident in the displacement in the C-terminal domains (Fig. 7 ▶ *a*). In addition to the difference in the hinge angle, the loops in proximity to the binding cavity also differ in EcAlr and CdAlr. Despite these differences, the active sites are well conserved and superpose quite well (Fig. 7 ▶ *a*). A closer comparison of the active sites reveals a conserved network of residues involved in interactions with either PLP or DCS (Figs. 7 ▶ *b* and 7 ▶ *c*). Additionally, Lys130 is carbamylated in both structures. Residues in the substrate entryway of EcAlr that are believed to be important for catalytic activity are conserved and superpose well with their counterparts from CdAlr (Fig. 6 ▶ *b*). The substrate entryway of EcAlr is less open than that of CdAlr because of the orientation of the equivalent residues to Arg312 and Asp172 that close off the entryway (Fig. 6 ▶ *b*). The major difference between the structures is that the hinge angle for EcAlr is substantially larger than that of CdAlr (Fig. 7 ▶ *a* and Table 3 ▶ *a*). Further studies are needed to determined whether this difference can be exploited for inhibitor design.

## 4. Conclusions   

The biochemical and structural studies of CdAlr presented here reveal that a mutant form of the enzyme has an unusually high catalytic activity despite an overall conserved fold with other members of the Alr family. The high catalytic activity of CdAlr_K271T_ is intriguing, and further studies of this mutant are needed to elucidate the structural basis for the observed difference. While the overall structure of CdAlr is similar to that of Alr from *E. coli*, further studies are required to determine whether the differences in the hinge region and the catalytic activity can be exploited for the design of CdAlr-specific inhibitors. The reported CdAlr crystal structures provide templates for future structure-based drug-design studies of the enzyme, with the ultimate goal of developing new antibiotics for multidrug-resistant *C. difficile*.

## Supplementary Material

PDB reference: CdAlr, complex with PLP, 4lus

PDB reference: complex with cycloserine, 4lut

PDB reference: K271T mutant, complex with PLP, 4luy

## Figures and Tables

**Table 1:** Statistics of data collection and model refinement Values in parentheses are for the highest resolution shell.

	CdAlr_PLP_ (4lus)	CdAlr_cycloserine_ (4lut)	CdAlr_K271T_ (4luy)
Data collection			
Space group	*P*2_1_	*P*2_1_2_1_2_1_	*P*2_1_
Unit-cell parameters (Å, °)	*a* = 85.2, *b* = 93.3, *c* = 107, β = 91	*a* = 57.87, *b* = 139.1, *c* = 144.93	*a* = 49.97, *b* = 154.33, *c* = 55.77, β = 113
Resolution limits (Å)	54.5–2.10 (2.20–2.10)	45.6–2.26 (2.33–2.26)	34.2–2.60 (2.74–2.60)
〈*I*/σ(*I*)〉	19.5 (4.9)	8.7 (1.8)	8.3 (1.9)
No. of observations	648855	204307	144493
No. of unique reflections	88688 (7550)	35962 (2623)	21924 (1477)
Multiplicity	7.5 (7.0)	5.7 (5.5)	6.2 (5.9)
*R*_merge_ † (%)	6.0 (37.4)	12.0 (82.7)	13.6 (54.8)
Completeness (%)	92.4 (52.9)	64.8 (55.6)	97.0 (90.1)
Average mosaicity (°)	0.98	1.42	0.88
Refinement			
*R*_cryst_ ‡ (%)	20.0 (25.8)	21.0 (30.2)	18.4 (24.7)
*R*_free_ § (%)	26.0 (30.5)	24.7 (31.4)	25.1 (35.5)
Correlation coefficient			
*F*_o_ − *F*_c_	0.953	0.939	0.948
*F*_o_ − *F*_c_ (free)	0.927	0.915	0.892
R.m.s. deviations			
Bond lengths (Å)	0.017	0.008	0.012
Bond angles (°)	1.942	1.352	1.577
Model composition			
Protein atoms	11792	5945	6018
Water atoms	277	196	100
Heterogen atoms	111	44	32
Ramachandran plot (%)			
Preferred regions	96.4	95.0	96.5
Allowed regions	2.9	4.0	2.6
Outliers	0.7	1.1	0.92

† *R*_merge_ = 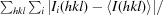 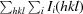, where *I*_*i*_(*hkl*) and 〈*I*(*hkl*)〉 are the intensity of the *i*th measurement and the mean intensity of the reflection with indices *hkl*, respectively.

‡ *R*_cryst_ = 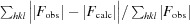, where *F*_obs_ are observed and *F*_calc_ are calculated structure-factor amplitudes.

§ The *R*_free_ set uses 5% of randomly chosen reflections.

**Table 2:** Comparison of kinetic parameters of selected Alrs NR, not reported.

		L-Ala→D-Ala			D-Ala→L-Ala		
Enzyme	Monomer MW	*K*_m_ (m*M*)	*V*_max_ (U mg^−1^ †)	*k*_cat_ (min^−1^)	*K*_m_ (m*M*)	*V*_max_ (U mg^−1^)	*k*_cat_ (min^−1^)
CdAlr	43306	17.6	82.1	3558	7	26.7	1155
CdAlr_K271T_	43279	13.8	4533	196532	5.4	1797	77905
GsAlr‡	43339	4.3	2550	NR	2.7	1400	NR
SlaAlr§	39888	0.4	NR	3800	0.4	NR	3300
MtbAlr¶	40721	4	5.3	200	NR	NR	NR
MsmAlr¶	41053	8.5	1000	4300	NR	NR	NR
EcAlr††	41316	1.0	NR	3239	0.3	NR	347

† One unit is defined as the amount of enzyme that racemizes 1 µmol of substrate per minute.

‡ Data from Inagaki *et al.* (1986 ▶).

§ Data from Noda *et al.* (2004 ▶).

¶ Data from Lee *et al.* (2013 ▶).

†† Data from Wu *et al.* (2008 ▶).

**(a):** Hinge angles of Alrs.

Protein	No. of atoms in αβ C-terminal domain	No. of atoms in N-terminal domain	Hinge angle (°)
CdAlr	1917	1106	123.50
EcAlr	1815	927	142.20
GsAlr	1970	1098	123.22
SlaAlr	1809	1031	124.76
SaAlr	1875	944	132.22
BaAlr	1904	1119	123.34
MtbAlr	1712	1001	136.16

**(b):** Intermolecular interactions across Alr dimer interfaces. BSA, buried surface area on monomer; MSA, overall monomer surface area; %BSA, total percentage of surface area that is buried at the dimer interface; NHB, number of hydrogen bonds; NSB, number of salt bridges; NMA, number of monomer amino-acid residues. Interactions were calculated using the *CCP*4 application *Protein Interfaces, Surfaces and Assemblies* (*PISA*; Krissinel & Henrick, 2007 ▶; Velankar *et al.*, 2010 ▶; Krissinel, 2010 ▶).

Protein	BSA (Å^2^)	MSA (Å^2^)	% BSA	NHB	NSB	NMA
CdAlr_K271T_	3387	17409	19	31	21	383
CdAlr_PLP_	3210	17411	19	35	21	382
CdAlr_cycloserine_	2755	17435	16	30	17	375
SlaAlr	2741	16124	17	34	6	382
GsAlr	3023	16499	19	46	18	382
MtbAlr	1934	15314	13	16	1	366
SaAlr	2456	16494	15	28	14	356
BaAlr	3572	16496	21	45	10	382
EcAlr	2818	16116	18	40	29	381
